# Gonorrhœal Arthritis: Its Diagnosis and Treatment

**Published:** 1907-06-08

**Authors:** 


### GONORRHŒAL ARTHRITIS: ITS DIAGNOSIS AND TREATMENT.

Gonorrhœal Arthritis occurs in many different degrees of severity. It may appear as an acute or sub-acute affection of one or of many joints; or as chronic pain, stiffness, and inflammatory thickening. Occasionally cases of passive effusion occur, most commonly in the knees, ankles, and shoulders. Perhaps the most serious cases are those in which many joints are affected and there is an acute or sub-acute febrile attack, for here the condition may be mistaken for rheumatic fever.

### Some Points in the Diagnosis.

Suspicion should always be aroused when joints outside the usual incidence of rheumatism—for example, the temporo-maxillary, sterno-clavicular, or sacro-iliac joints—are involved. Inflammation and thickening outside the capsule of the joint, œdema of the subcutaneous tissues, and thickening and tenderness of the bones, as if from periostitis, should also suggest a gonococcal origin. Again, if local symptoms and fever persist or recur in spite of the administration of salicylates, doubt of the "rheumatic" nature of the attack may well arise, and obviously in all doubtful cases it is wise to make certain by investigation directed to the discovery of a urethral or vaginal discharge. Even then there are opportunities for error.

### Its Onset.

Gonorrhœal arthritis rarely develops until some weeks after the primary affection, and it may not appear for months. Hence the original gonorrhœal discharge may in the male have been reduced to a slight "gleet," and even in the female there may be no conclusive evidence of a specific vaginitis in the urethra or in the vagina; such evidence, however, may be found in the glands of Bartholin and in the canal of the cervix uteri. Thus it is a sound rule of practice that what seems to be "rheumatism" may really be an arthritis following gonorrhœa. With this rule clearly before him the practitioner will be saved from many serious blunders.

### The Treatment.

The first aim of treatment is to cure the gonorrhœa, on which the whole train of subsequent symptoms depends. So long as there is permitted further opportunity for absorption of the poison, so long will joint conditions persist. Almost every practitioner has his own favourite remedy for the local treatment of gonorrhœa, and it may be surmised that the secret of success depends less on the specific virtues of the remedy than on the thoroughness of its application. The writer of this note prefers either potassium permanganate (1 in 1,000) or zinc chloride (1 grain to the ounce), and the frequency of application is determined by the abundance of the discharge. Always before injecting the prescribed solution a quantity of warm water should be used to cleanse the mucous membrane, and thus to permit the remedial agent to come into contact with the site of the mischief; the necessity for this procedure is especially urgent in the case of women. A second demand in the treatment of gonorrhœal arthritis is maintenance of the patient's general health and vigour. Here is one of the reasons why an erroneous diagnosis of rheumatism may carry such serious consequences. For the restricted diet which, wisely or unwisely, is often ordered in cases of rheumatism is most unsuitable for the subjects of gonorrhœal arthritis. These patients are depressed both mentally and physically, and an important element in conquering the disease is the restoration of the general health. Probably quinine with potassium bicarbonate and citrate, all in full doses, is the best medicinal agent so long as there is much fever. But when the temperature is normal or approximately normal the quinine may be seconded by such tonics as cod-liver oil, hypophosphites and strychnine, and a generous dietary should be permitted.

### A Third Point.

The third principle of treatment which demands recognition is fixation of the affected joints, if necessary under a general anæsthetic. This may be combined with moderate pressure, and should be interrupted at frequent intervals by passive movement with a view to prevent stiffening of the joints. Light splints and bandages, and even indiarubber bandages, may be used for these purposes, but some prefer plaster of Paris. In any event careful padding of the joints should be adopted, as there is usually much muscular wasting, and this, together with the local tenderness, make adequate protection from excessive pressure of great moment. As the joint conditions improve massage, passive movements, exercises, and electricity may help to restore strength and freedom of movement. The same is true of hot air and electric light baths, and indeed these have a field of utility even when the arthritis is still active. Puncture or incision of a joint are rarely needed in gonorrhœal arthritis; nor is it usually necessary to incise the integument in cases where there is inflammatory swelling of the subcutaneous tissue in the neighbourhood of the joint—a condition, by the way, that is sometimes mistaken for cellulitis.

### The Use of Serum.

Lastly must be mentioned the use of anti-streptococcic serum. Probably the toxins absorbed in gonorrhœa are not merely those of the gonococcus, but also of various other organisms. At all events it is a fact that the injection of a polyvalent anti-streptococcic serum has produced much benefit in acute and sub-acute cases. A dose of 20 cubic centimetres may be given and followed by 10 c.c. daily for three or four days. In very severe and urgent cases two doses may be given daily. Dr. Soltau Fenwick and Dr. Porter Parkinson report decidedly encouraging results.

